# Gender‐specific outcomes of low‐dose computed tomography screening for lung cancer detection: A retrospective study in Chinese never‐smoker population

**DOI:** 10.1002/cam4.70184

**Published:** 2024-09-29

**Authors:** Huihong Wang, Jicheng Xie, Yahong Chen, Jiang Jin, Meixian Zhang, TaoHsin Tung, Youzu Xu

**Affiliations:** ^1^ Department of Respiratory and Critical Care Medicine Taizhou Hospital of Zhejiang Province affiliated to Wenzhou Medical University Linhai Zhejiang China; ^2^ Department of radiology Taizhou Hospital of Zhejiang Province affiliated to Wenzhou Medical University Linhai Zhejiang China; ^3^ Health Management Center Taizhou Hospital of Zhejiang Province affiliated to Wenzhou Medical University Linhai Zhejiang China; ^4^ Department of Thoracic Surgery Taizhou Hospital of Zhejiang Province affiliated to Wenzhou Medical University Linhai Zhejiang China; ^5^ Public Laboratory Taizhou Hospital of Zhejiang Province affiliated to Wenzhou Medical University Linhai Zhejiang China

**Keywords:** gender‐specific outcomes, low‐dose computed tomography (LDCT), lung cancer, pulmonary nodule

## Abstract

**Objectives:**

Low‐dose computed tomography (LDCT) has emerged as a pivotal tool for detecting lung cancer among ever‐smokers. This study aims to evaluate the gender‐specific outcomes of LDCT screening within the Chinese never‐smoking population.

**Methods:**

We conducted a single‐center, retrospective cohort study, which analyzed LDCT screening outcomes for 42,018 asymptomatic participants. Specifically, we focused on assessing gender‐specific differences in the prevalence of pulmonary nodules, and the incidence of lung cancer diagnosis among never‐smokers.

**Results:**

Among the 42,018 eligible participants, 41.50% were females and 58.50% were males. Most participants were non‐smokers (77.57%), with a significantly higher proportion of non‐smokers among females than males (99.33% vs. 62.14%). Pulmonary nodules were identified in 2.66% of participants, with a higher prevalence in females (2.99%) than males (2.43%) (*p <* 0.001). Non‐smoking females had a higher incidence of positive nodules than non‐smoking males (2.98% vs. 2.38%, *p <* 0.001). Invasive biopsies were performed in 334 individuals with nodules, confirming lung cancer in 258 cases. The majority of these cancer cases were non‐smokers (212), with non‐smoking females showing a higher incidence (0.85%) compared to males (0.43%) (*p* < 0.001). There was no significant difference in the false‐positive rates between non‐smoking females (0.14%) and males (0.13%). Multivariate analysis showed that never‐smoking women were more likely to undergo biopsies (OR 1.65, *p =* 0.0016) and had a higher, though not statistically significant, probability of lung cancer diagnosis (OR 1.84, *p* = 0.06).

**Conclusion:**

This study elucidates sex‐based differences within the Chinese population, revealing a higher prevalence of pulmonary nodules and lung cancers among non‐smoking females. These findings offer valuable reference for both clinical practice and future research initiatives.

## 1 INTRODUCTION

Lung cancer remains the second most prevalent cancer and the leading cause of cancer‐related deaths worldwide.^1^, ^2^, ^3^ In China, it's a leading cause of both cancer‐related mortality and new cases incidence.^4^, ^5^, ^6^, ^7^, ^8^, ^9^, ^10^ Despite medical advancements, the 5‐year survival rate in China only modestly increased from 16.1% in 2003–2005 to 19.7% in 2012–2015.^11^ This limited improvement is mainly due to the challenges in early diagnosis.

Low‐dose computed tomography (LDCT) has been identified as an effective tool for early detection of lung cancer in heavy smokers, evidenced by trials like NLST and NELSON.^12^, ^13^, ^14^, ^15^, ^16^, ^17^ Building on these findings, the US Preventive Services Task Force (USPSTF) recommends LDCT screening for high‐risk smokers.^18^ Following the NLST results, along with China's experience,^19^ China has established guidelines that recommend annual LDCT screenings for individuals aged 50–74 years, with at least 20 years of smoking history.^20^, ^21^, ^22^, ^23^, ^24^, ^25^

While LDCT screening is well‐established for heavy smokers, it may not fully address high‐risk groups, particularly non‐smoking women.^26^, ^27^ A recent study showed that LDCT effectively identifies lung nodules in never‐smokers, advocating for more tailored management strategies in Asian never‐smokers.^26^ However, this study did not delve into gender‐specific differences. From 2000 to 2014, lung cancer rates in never‐smoking females more than doubled, representing over 85% of female lung cancer cases.^8^, ^28^ In the United States, young females now exhibit higher lung cancer incidence rates than young males.^29^ In China, about 22% of lung cancer patients are never‐smokers, predominantly women (61%).^30^, ^31^ Remarkably, the incidence of lung cancer among Chinese women (22.8/100,000) is comparable to, or even exceeds, that in several Western European countries like France (22.5/100,000), despite much lower smoking rates among Chinese women.^1^ These trends suggest that current inclusion criteria may not adequately cover all high‐risk individuals, especially non‐smoking Chinese women.

While LDCT screening can offers benefits in reducing lung cancer mortality, it also introduces potential adverse effects such as radiation risks, and false‐positive results, which lead to unnecessary invasive procedures.^3^, ^32^, ^33^, ^34^ Several studies indicate that for individuals under 50 years old, the risks may outweigh the benefits.^35^ The false‐positive rate of never‐smoking Chinese women undergoing LDCT screening requires targeted investigation.

To address these gaps, we conducted a retrospective study on asymptomatic individuals who underwent LDCT lung cancer screening in China. This study specifically focused on gender disparities among never‐smokers, analyzing the incidence of pulmonary nodules, biopsy rates, and diagnostic outcomes in a real‐world Chinese setting.

## 2 MATERIALS AND METHODS

### 2.1 Study design and participants

We conducted a single‐center, retrospective cohort study at the Health Management Center of Taizhou Hospital, Zhejiang Province. The study was approved by the Ethics Review Committee of Taizhou Hospital. Written informed consent was obtained from the participants. Our study cohort comprised healthy individuals who participated in LDCT screening from January 1, 2017, to December 31, 2020. Eligible participants met the following inclusion criteria: (1) aged 20–80 years, and (2) No significant clinical symptoms at the time of baseline screening. We excluded individuals with (1) a history of lung cancer, or (2) undocumented smoking history.

### 2.2 Procedures

Screening was performed using a 32‐slice multi‐detector computed tomography with a GE LightSpeed VCT scanner. Participants were positioned in the supine position with both arms raised and placed beside the head to ensure full exposure of the chest. Scanning was performed at the end of deep inspiration, covering the entire lung from apices to costophrenic angles.

Scanning parameters included helical CT volumetric scanning with a minimum collimation of 0.55 mm. Tube voltage ranged from 100 to 120 kVp depending on individuals' weight, with a tube current ranged from 30 to 50 mAs and a total radiation exposure dose limiting to 1 mSv. The lung window settings were at −600 Hounsfield units (Hu) with a width of 1600 Hu, and the mediastinal window settings were at 30 Hu with a width of 400 Hu. LDCT images were reconstructed and analyzed using computer‐aided detection software (Shanghai Haohua Technology Co Ltd.), with a manual review by an expert (Jicheng Xie) to guarantee accuracy.

For participants who underwent multiple LDCT scans, the number of scans and the time of the last scan were recorded. For patients who had invasive procedures, the follow‐up CT scans were tracked until the time of surgery.

Consistent with NLST protocols,^16^ lung nodules with a maximum diameter ≥4 mm were selected based on CT results for further analysis. Nodule evaluation was carried out by a pulmonologist (Huihong Wang), a radiologist (Jicheng Xie) and a thoracic surgeon (Jiang Jin). Due to the update of the American College of Radiology Lung‐RADS from version 1.0 (2014)^36^ to version 1.1 (2019)^37^ during our study's enrollment period, we re‐assessed the LDCT images of participants who were enrolled before 2019 using the revised criteria of version 1.1. This ensured that all nodules in this study were assessed uniformly according to the Lung‐RADS version 1.1 criteria.^37^ In cases of multiple nodules, the highest‐risk nodule was selected for analysis. A representative case of multiple nodules has been included in the supplementary material. Additionally, the methodology of the biopsy procedure, as well as details of the subsequent CT scan of the lung, are detailed.

For Lung‐RADS categories 3 and 4, nodule size, location, and characteristics were recorded. Pathological confirmation of positive screening results was obtained through open thoracotomy, video‐assisted thoracic surgery (VATS), percutaneous needle biopsy, or bronchoscopic biopsy methods. Lung cancer staging was performed according to the eighth edition of the International Lung Cancer TNM staging system.^38^

### 2.3 Univariate and multivariate cox analysis

Univariate and multivariate Cox proportional hazards regression analyses were conducted on non‐smokers to identify factors associated with the incidence of positive nodules, biopsy rates, and lung cancer detection rates. The analyses used the R package “survival” and included variables such as age and gender. Hazard ratios (HRs) with 95% confidence intervals (CIs) were calculated for each variable. The results were illustrated using forest plots generated with the R package “forestplot.”

### 2.4 Survival analysis

Survival data were collected up to March 30, 2024. Survival analysis was conducted using the R package “survival.” Kaplan–Meier survival curves were generated to estimate overall survival and differences between groups were evaluated using the log‐rank test. The survival curves were visualized using the R package “survminer.”

### 2.5 Statistical analysis

Participant characteristics were presented as frequencies (%) for categorical variables and as mean ± SD for continuous variables. Comparative analysis between sexes within the never‐smokers or ever‐smokers group was performed. Pearson Chi‐squared tests were applied for categorical variables, and *t*‐tests for continuous variables. *p* ≤ 0.05 was considered statistical significance. All statistical analyses were performed using R v4.2.2.

## 3 RESULTS

### 3.1 Participant demographics in LDCT screening

**FIGURE 1:**
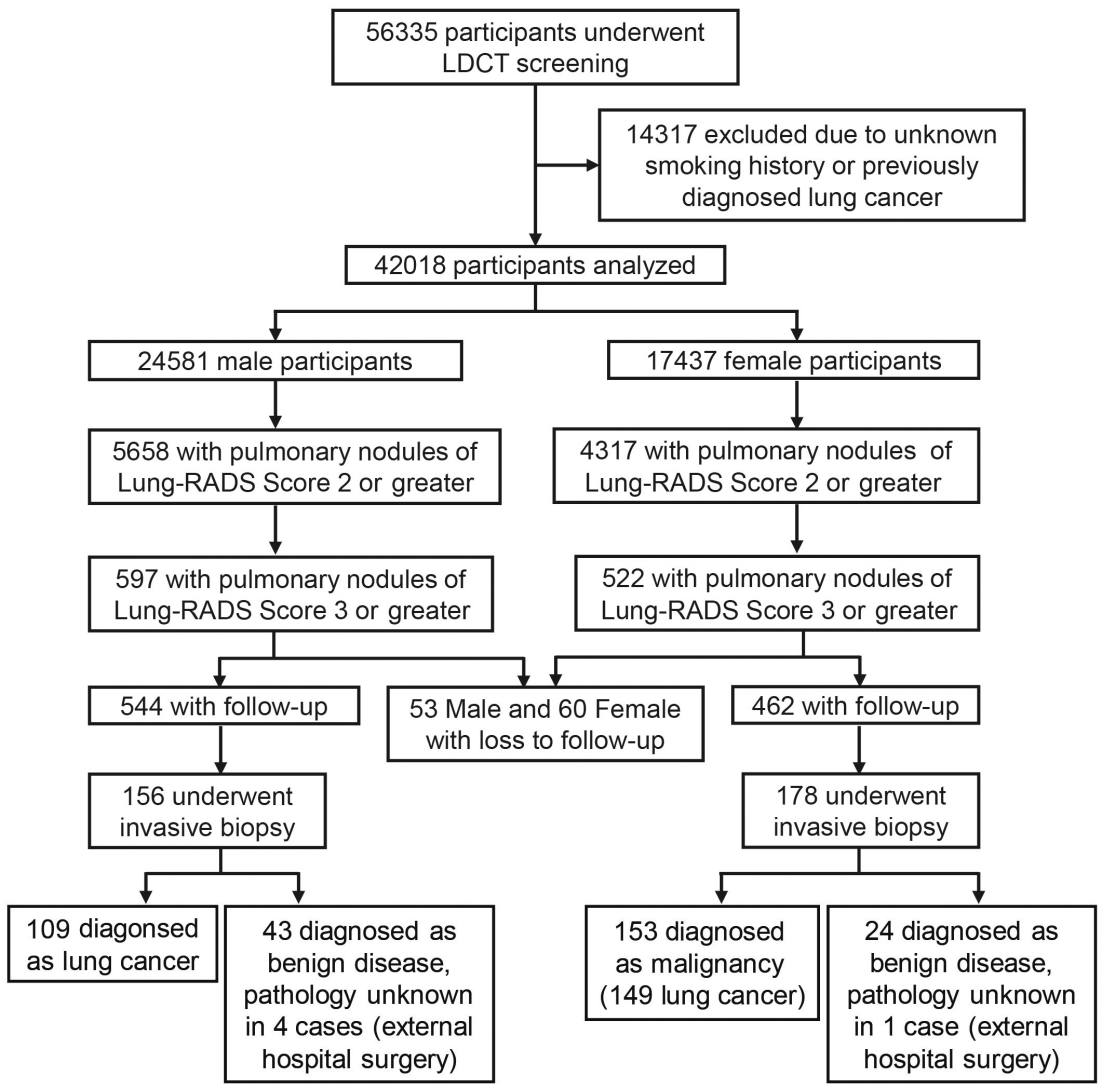
Flowchart of participant inclusion for low‐dose computed tomography (LDCT) screening.

Figure 1 provides the flowchart that illustrates the inclusion of the participants in our study. Over the study period, a total of 56,335 individuals underwent LDCT screening. After excluding 14,317 individuals with an unknown smoking history or a previous lung cancer diagnosis, 42,018 participants were eligible for analysis. Table 1 provides the baseline demographic of all participants, comprising 17,437 females (41.50%), and 24,581 males (58.50%). Never‐smokers constituted 77.57% (32,595/42,018) of all participants, with females having a significantly higher representation (99.33%, 17,320/17,437) than males (62.14%, 15,275/24,581) (*p <* 0.001).

**TABLE 1:** Demographics of participants who underwent low‐dose computed tomography (LDCT) screening.

	Total (%)	Male (%)	Female (%)	*p*‐value
Subjects	42,018	24,581	17,437	
Age range				
20–29 years	4761 (11.33)	3049 (12.40)	1712 (9.82)	<0.001
30–39 years	8327 (19.82)	5100 (20.75)	3227 (18.51)	
40–49 years	10,981 (26.13)	6374 (25.93)	4607 (26.42)	
50–59 years	10,781 (25.66)	6149 (25.02)	4632 (26.56)	
60–69 years	5234 (12.46)	2798 (11.38)	2436 (13.97)	
70–80 years	1934 (4.60)	1111 (4.52)	823 (4.72)	
Ever‐smoker	9423 (22.43)	9306 (37.86)	117 (0.67)	<0.001
BMI kg/m^2^	23.76 ± 3.29	25.37 ± 3.26	23.22 ± 3.21	<0.001
Lung‐RADS category (at baseline LDCT)				
1 or S	32,043 (76.26)	18,923 (76.98)	13,120 (75.24)	<0.001
2	8856 (21.08)	5061 (20.59)	3795 (21.76)	
3	674 (1.60)	371 (1.51)	303 (1.74)	
4A	156 (0.37)	83 (0.34)	73 (0.42)	
4B	55 (0.13)	33 (0.13)	22 (0.13)	
4X	234 (0.56)	110 (0.45)	124 (0.71)	
Nodules of Lung‐RADS Score 3 and 4	1119 (2.66)	597 (2.43)	522 (2.99)	<0.001

Abbreviation: Lung‐RADS, Lung imaging reporting and data system.

### 3.2 Non‐smoking females exhibit higher pulmonary nodule prevalence

During this study, 1119 participants (2.66%, 1119/42,018) were identified with pulmonary nodule, classified as lung‐RADS category 3 or 4 by LDCT (Table 1). Among these, about 60% (674/1119) were categorized as lung‐RADS 3, while the remaining 40% (445/1119) were classified as lung‐RADS category 4, including subcategories 4A, 4B, and 4X (*p <* 0.001) (Table S1).

We found that the prevalence of positive nodules was significantly higher in females (2.99%, 522/17,437) than in males (2.43%, 597/24,581) (*p <* 0.001) (Table 1). Considering the extremely low rate of smokers in women, we further compared the prevalence of nodules by gender in the non‐smoker or ever‐smoker subgroups. Among non‐smokers, the ratio of positive nodules was significantly higher in women (2.98%, 517/17,320) than in men (2.38%, 363/15,275) (*p <* 0.001) (Table 2). While among the ever‐smokers with positive results, there was no significant difference in the rate of pulmonary nodules between females (4.27%, 5/117) and males (2.51%, 234/9306) (*p =* 0.225) (Table 3).

**TABLE 2:** Comparison of detection rates in never‐smoking females versus males.

	Total (%)	Male (%)	Female (%)	*p*‐value
Subjects	32,595	15,275	17,320	
Nodules of Lung‐RADS Score 3 and 4	880 (2.70)	363 (2.38)	517 (2.98)	<0.001
Lung cancer	212 (0.65)	65 (0.43)	147 (0.85)	<0.001
Early‐stage	190 (0.58)	53 (0.35)	137 (0.79)	<0.001
False‐positive	44 (0.13)	20 (0.13)	24 (0.14)	0.971

**TABLE 3:** Comparison of detection rates in smoking females versus males.

	Total (%)	Male (%)	Female (%)	*p*‐value
Subjects	9423	9306	117	
Nodules of Lung‐RADS Score 3 and 4	239 (2.54)	234 (2.51)	5 (4.27)	0.225
Lung cancer	46 (0.49)	44 (0.47)	2 (1.71)	0.215
Early‐stage	27 (0.29)	25 (0.27)	2 (1.71)	0.043
False‐positive	23 (0.24)	23 (0.25)	0	NA

Surprisingly, the incidence of pulmonary nodules was 2.54% (239/9423) among ever‐smokers and 2.70% (880/32,595) among non‐smokers, with no significant difference (*p* = 0.404) (Table S2). To control for gender, we analyzed the positive nodule detection rates by smoking status for men and women separately. In men, the incidence was 2.51% (234/9306) for ever‐smokers and 2.38% (363/15,275) for non‐smokers, showing no significant difference (*p* = 0.523) (Table S3). In women, the rates were 4.27% (5/117) for ever‐smokers and 2.98% (517/17,320) for non‐smokers, also showing no significant difference (*p* = 0.587) (Table S4).

These results indicate that although there is no gender difference in ever‐smokers, the incidence of positive nodules is significantly higher in non‐smoking females compared to non‐smoking males. Therefore, non‐smoking females should be specifically considered in LDCT screening.

### 3.3 Females with positive nodules undergo more biopsies

Based on follow‐up results, invasive biopsies were performed in 334 individuals (33.20%, 334/1006) among those with pulmonary nodules. Females exhibited a significantly higher ratio of underwent invasive biopsies (38.53%, 178/462) compared to males (28.68%, 156/544) (*p =* 0.005) (Table S1).

Among those who underwent biopsies, the frequency of LDCT screenings and the growth of nodules were relatively comparable between females and males before the biopsy, showing no significant difference (*p =* 0.080). Out of 334 biopsied specimens, 262 were classified as malignancy, with 258 confirmed as lung cancer, accounting for 77.25% (258/334) of the total biopsied participants. The remaining four cases were identified as either metastatic carcinoma or lymphoma (Table S5).

### 3.4 Non‐smoking females have higher lung cancer incidence

Of the 258 patients diagnosed with lung cancer, 149 were females and 109 were males. The characteristics of these patients are shown in Table 4. Notably, only 2 of the 149 women diagnosed with lung cancer had a history of smoking, while the remaining 147 were non‐smokers. It prompted us to investigate the influence of gender on lung cancer incidence in never‐smokers or ever‐smokers.

**TABLE 4:** Characteristics of diagnosed lung cancers.

	Total (%)	Male (%)	Female (%)	*p*‐value
Subjects	258	109	149	
Smoking status				
Never‐smokers	212 (82.17)	65 (59.63)	147 (98.66)	<0.001
Ever‐smoker	46 (17.83)	44 (40.37)	2 (1.34)	<0.001
Age range				0.684
20–29 years	11 (4.26)	3 (2.75)	8 (5.37)	
30–39 years	21 (8.14)	9 (8.26)	12 (8.05)	
40–49 years	52 (20.16)	19 (17.43)	33 (22.15)	
50–59 years	76 (29.46)	31 (28.44)	45 (30.2)	
60–69 years	64 (24.81)	31 (28.44)	33 (22.15)	
70–80 years	34 (13.18)	16 (14.68)	18 (12.08)	
Nodule type				0.013
Solid	78 (30.23)	43 (39.45)	35 (23.49)	
Part‐solid	177 (68.60)	64 (58.72)	113 (75.84)	
Cavitary	3 (1.16)	2 (1.83)	1 (0.67)	
Type of biopsy				<0.001
VATS	238 (92.25)	94 (86.24)	144 (96.64)	0.004
Percutaneous needle biopsy	6 (2.33)	2 (1.83)	4 (2.68)	
Bronchoscopic biopsy	13 (5.04)	13 (11.93)	0	<0.001
Biopsy of pleural effusion	1 (0.39)	0	1 (0.67)	
Cancer histology				<0.001
Adenocarcinoma	240 (93.02)	92 (84.40)	148 (99.33)	<0.001
Adenosquamous (Squamous)	10 (3.88)	10 (9.17)	0	0.001
Other nonsmall cell carcinoma	3 (1.16)	2 (1.83)	1 (0.67)	
Small cell carcinoma	5 (1.94)	5 (4.59)	0	
Unkown	5 (1.94)	4 (3.67)	1 (0.67)	
Lung cancer staging				0.003
IA	217 (84.11)	81 (74.31)	136 (91.28)	0.001
IB	8 (3.10)	5 (4.59)	3 (2.01)	
II	9 (3.49)	5 (4.59)	4 (2.68)	
III	9 (3.49)	8 (7.34)	1 (0.67)	
IV	11 (4.26)	6 (5.50)	5 (3.36)	
NA for TNM staging	4 (1.55)	4 (3.67)	0	
Initial treatment				<0.001
Surgery	216 (83.72)	81 (74.31)	135 (90.60)	0.001
Surgery+adjuvant therapy	22 (8.53)	13 (11.93)	9 (6.04)	0.148
Targeted therapy and or chemotherapy and or radiotherapy	16 (6.20)	12 (11.01)	4 (2.68)	0.013
Supportive care only	2 (0.78)	1 (0.92)	1 (0.67)	
Unknown	2 (0.78)	2 (1.83)	0	

Abbreviation: VATS, video‐assisted thoracic surgery.

Among the 212 never‐smoking patients, the rate of lung cancer diagnosis was significantly higher in never‐smoker women (0.85%, 147/17320) compared to never‐smoker men (0.43%, 65/15275) (*p <* 0.001) (Table 2). At the same time, no significant gender difference was observed among ever‐smokers (*p =* 0.215) (Table 3).

Within the study cohort, 67 individuals were diagnosed with benign nodules. Across the non‐smoker subgroup, there was no significant difference between genders (*p =* 0.773) (Table 2). Further details on these false‐positive cases are provided in Table S6.

These results show a significantly higher lung cancer incidence in non‐smoking females compared to males. The false‐positive rate in never‐smoking women is not elevated despite a higher detection rate. These findings underscore the importance of prioritizing non‐smoking women in LDCT screening.

### 3.5 Smoking increases lung cancer risk in men but not in women

We also examined the association between smoking and lung carcinogenesis. Ever‐smokers constituted 0.49% (46/9423) of cases, while non‐smokers accounted for 0.65% (212/32595), showing no significant difference (*p =* 0.089). To assess the effectiveness of screening, we compared follow‐up scans and lung cancer detection patterns between smokers and non‐smokers. Among lung cancer patients, 26.09% (12/46) of smokers were detected at the first LDCT scan, and 73.91% (34/46) were detected at subsequent follow‐up scans. Similarly, 75.47% (160/212) of non‐smokers were detected at follow‐up visits, and only 24.53% (52/212) were detected at the initial scan. This suggests that follow‐up of non‐smokers is also necessary.

To control for gender, we analyzed the cancer incidence by smoking status for men and women separately. In men, the lung cancer prevalence was significantly higher in ever‐smokers (0.70%, 65/9306) compared to non‐smokers (0.29%, 44/15,275), suggesting smoking as a risk factor for men (Table S3). While for women, the cancer rates were 1.71% (2/117) for ever‐smokers and 0.85% (147/17,320) for non‐smokers, with no significant difference (*p* = 0.614) (Table S4).

This analysis indicates that smoking is a risk factor for men but not for women, suggesting other risk factors may influence women. These findings also emphasize the need for regular follow‐up in non‐smokers.

### 3.6 Non‐smoking women show higher early‐stage lung cancer rates

Since LDCT screening focuses on early‐stage lung cancer and the majority of lung cancer cases in our cohort were early‐stage (217/258, 84.11%), we first stratified participants by smoking status and then compared the detection rates of stage IA lung cancer across genders within each group of never‐smokers and ever‐smokers. Among non‐smokers, the proportion of stage IA lung cancer was significantly higher in women (0.79%, 137/17,320) than in men (0.35%, 53/15,275) (*p* < 0.001) (Table 2). Among ever‐smokers, the proportion of stage IA lung cancer was also higher in women (1.71%, 2/117) than in men (0.27%, 25/9306) (*p* = 0.043) (Table 3). Additionally, within the non‐smoking cancer cohort, the proportion of stage IA lung cancer was significantly higher in women (93.20%, 137/147) than in men (81.54%, 53/65) (*p* = 0.014). These results demonstrate the pronounced effectiveness of LDCT screening in non‐smoking women, particularly for early‐stage lung cancer. The higher incidence of both lung cancer and early‐stage cancer in non‐smoking women underscores the necessity of LDCT screening for this group.

### 3.7 Regression analysis reveals higher lung cancer detection rates in never‐smoking women

**FIGURE 2:**
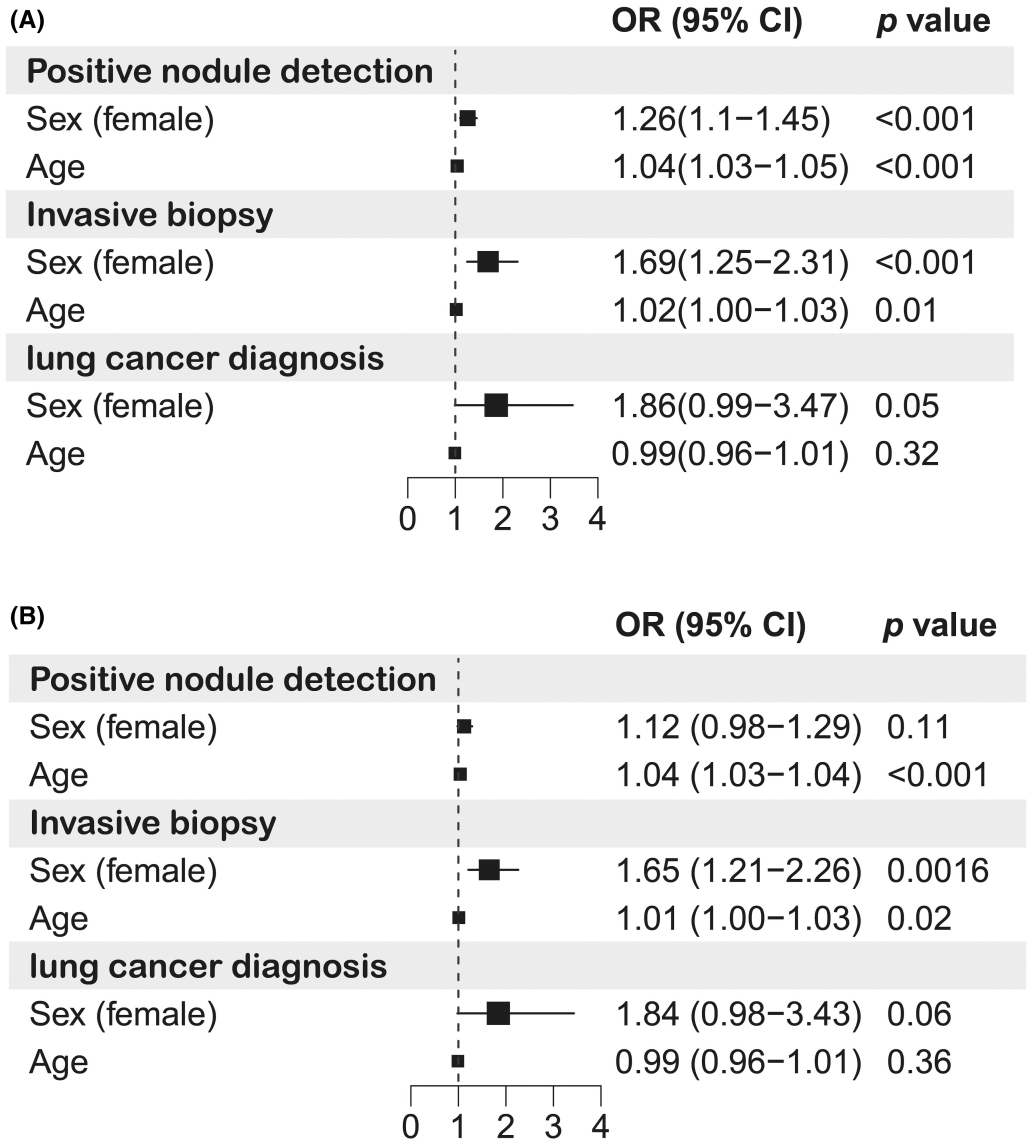
Forest plots of univariate (A) and multivariate (B) regression analyses of low‐dose computed tomography (LDCT) screening outcomes in never‐smokers. OR, odds ratio, 95% CI, 95% confidence intervals.

**FIGURE 3:**
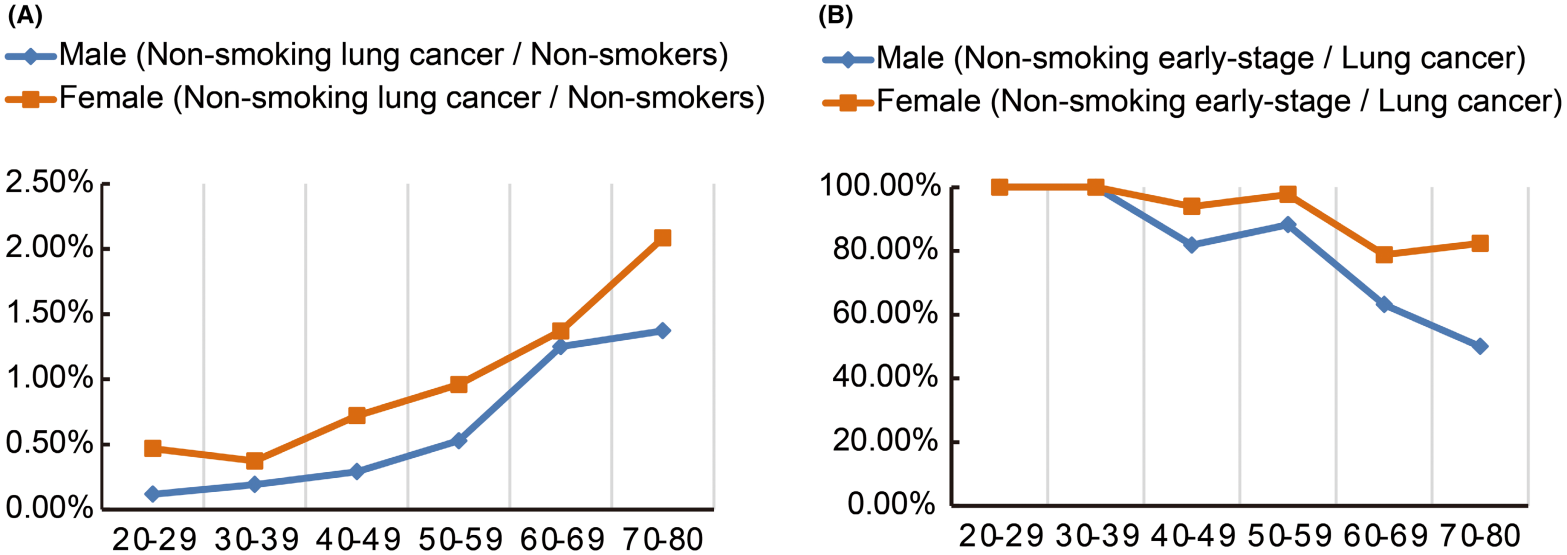
Comparative analysis of (A) overall lung cancer detection rates and (B) Stage IA lung cancer detection rates between never‐smoking females and males across different age groups.

To explore the relationship between demographic factors and clinical outcomes, we performed univariate and multivariate regression analyses among never‐smoking participants (Figure 2). Univariate analysis revealed that never‐smoking women were significantly more likely to have a lung nodule (OR 1.26, 95% CI 1.10–1.45, *p <* 0.001), undergo an invasive biopsy (OR 1.69, 95% CI 1.25–2.31, *p <* 0.001), and be diagnosed with lung cancer (OR 1.86, 95% CI 0.99–3.47, *p =* 0.05) (Figure 2A). Notably, never‐smoking females exhibited higher cancer detection rates across all age groups compared to never‐smoking males (Figure 3A,B, Table 5).

**TABLE 5:** Gender‐based comparison of lung cancer detection rates among non‐smokers across age groups.

Character‐istics	Male			Female		
	Total (%)	Lung cancer (%)	Stage IA (%)	Total (%)	Lung cancer (%)	Stage IA (%)
Total	15,275	65 (0.43%)	53 (81.54%)	17,320	147 (0.85%)	137 (93.20%)
Age range						
20–29 years	2534	3 (0.12%)	3 (100%)	1708	8 (0.47%)	8 (100%)
30–39 years	3646	7 (0.19%)	7 (100%)	3215	12 (0.37%)	12 (100%)
40–49 years	3775	11 (0.29%)	9 (81.82%)	4587	33 (0.72%)	31 (93.94%)
50–59 years	3218	17 (0.53%)	15 (88.24%)	4586	44 (0.96%)	43 (97.73%)
60–69 years	1519	19 (1.25%)	12 (63.16%)	2408	33 (1.37%)	26 (78.79%)
70‐80 years	583	8 (1.37%)	4 (50.00%)	816	17 (2.08%)	14 (82.35%)
*r*‐value		0.884	−0.873		0.884	−0.725
*p*‐value		0.004	0.002		0.007	0.036

After adjusting for multiple variables, women remained significantly more likely to undergo a biopsy (OR 1.65, 95% CI 1.21–2.26, *p =* 0.0016) and had a higher, though not statistically significant, probability of being diagnosed with lung cancer (OR 1.84, *p =* 0.06) (Figure 2B). These findings highlight the considerable impact of gender on clinical outcomes of LDCT screening, particularly the likelihood of receiving an invasive biopsy.

### 3.8 Survival outcomes in non‐smoking lung cancer patients

Despite the immaturity of the data, we analyzed the survival outcomes of lung cancer patients by gender. The limited number of female smokers (only two) precluded meaningful gender comparisons among ever‐smokers, so we focused on non‐smokers. In this group, we found no significant difference in survival between males and females (Figure S2A). Most patients with early‐stage lung cancer remain alive, with similar survival outcomes for non‐smoking males and females (Figure S2B). Among the 27 non‐smokers with advanced lung cancer, only one male is still alive, and no significant gender differences in survival were observed (Figure S2C).

## 4 DISCUSSION

This study involved 42,018 participants in LDCT lung cancer screening, revealing significant gender‐specific observations in China. Among the participants, non‐smoking women demonstrated higher rates of being categorized with lung RADS 3 or 4 and underwent more invasive biopsy procedures compared to non‐smoking men. Of the diagnosed cancers, women were predominantly presented with adenocarcinoma and were more often diagnosed at an early stage (stage IA), resulting in a higher rate of surgical resection, also highlighting the efficacy of LDCT screening in females. The majority of the women diagnosed with lung cancer were non‐smokers (147 out of 149), suggesting alternative etiology of lung cancer in the Chinese female population that is not related to smoking. Additionally, women exhibited higher detection rates of lung cancer across all age groups in our study, underscoring the need for gender‐specific screening strategies and further research into the unique patterns of lung cancer in women.

While existing large randomized LDCT screening trials have focused predominantly on heavy smokers, our research unveils the potential advantages in Chinese women, who demonstrate an exceptionally low smoking prevalence (0.67%). A recent meta‐analysis of 13 Asian studies showed that Asian female never‐smokers had a lung cancer incidence comparable to that of Asian male ever‐smokers.^39^ Previous studies have shown that over 80% of lung cancers in Western countries are smoking‐related, whereas in China, lung cancer risk factors exhibit greater complexity.^40^ Non‐smoking‐related factors, such as severe air pollution and biofuel use, play a more crucial role in lung cancer development in China,^41^ with Chinese women frequently exposed to smoke from charcoal used in heating and cooking. Recent revisions to the Chinese LDCT screening guidelines for lung cancer now consider additional risk factors beyond smoking, such as carcinogen exposure and passive smoking, but still do not explicitly include this high‐risk group of women.^25^

Although it is often suggested that extensive LDCT screening might lead to unnecessary invasive biopsies for benign nodules, our study found no significant difference in the false‐positive rate between genders. Remarkably, within the biopsy subset, women demonstrated a lower rate of false positives. To reduce invasive treatments for benign nodules, meticulous monitoring and assessment of potential non‐cancerous conditions are essential, even in cases classified as Lung‐RADS category 4. In diagnostic procedures, prioritizing less invasive diagnostic methods before surgical interventions could decrease the risks and burdens of unnecessary surgeries. The strength of our study lies in its large sample size and representation of an asymptomatic Chinese population undergoing LDCT screening in a real‐world, tertiary medical center setting. The comprehensive data on screening outcomes and diagnostics offer unique insights, contributing to further discussions on LDCT screening among Chinese females.

However, our study has limitations. It was a retrospective cohort study conducted from a single center, and the strategies for LDCT screening and follow‐ups were not strictly controlled, which led to some loss of follow‐up among individuals with positive screening results. Our cohort primarily consisted of individuals from Taizhou, a city in eastern China. Prior research has illustrated regional disparities in lung cancer incidence across China, with populations in the eastern regions, particularly females, exhibiting an elevated risk compared to western regions.^8^, ^10^, ^42^ This geographical bias could potentially lead to an overestimation of the benefits of LDCT screening for Chinese women. To substantiate our conclusions, it is essential to conduct expansive, multicentric, and cross‐regional studies. This approach would provide a more comprehensive understanding of the benefit of LDCT screening across diverse demographic and geographical landscapes in China. Furthermore, the study wasn't designed to evaluate the effectiveness of lung cancer screening over non‐screening in never‐smoking females, and therefore our results do not directly advocate for general LDCT screening in this population. A large trial with a control group of unscreened females and an associated cost–benefit analysis is needed for more conclusive evidence. Moreover, data on exposures to possible risk factors for lung cancer such as second‐hand smoke, charcoal used in heating and cooking, or air pollution were not available, which are crucial for assessing lung cancer screening in never‐smoking females. Additionally, the hospital‐based design may not fully represent the general population, though we include only asymptomatic adults undergoing health check‐ups to minimize selection bias.

## 5 CONCLUSIONS

In conclusion, LDCT screening in Chinese women resulted in a significant detection of nodules with positive results, which led to invasive diagnostic procedures. The subsequent lung cancer detection rate was much higher in non‐smoking females than in non‐smoking males, with false‐positive rates comparable to males. Our findings emphasize the necessity for developing gender‐specific screening protocols and nodule management strategies.

## AUTHOR CONTRIBUTIONS

**Huihong Wang:** Writing – original draft (equal). **Jicheng Xie:** Writing – original draft (equal). **Yahong Chen:** Writing – original draft (supporting). **Jiang Jin:** Investigation (supporting); resources (supporting).

## CONFLICT OF INTEREST STATEMENT

The authors have no conflict to declare.

## ETHICS STATEMENT

The study was approved by the Ethics Review Committee of Taizhou Hospital. Written informed consent was obtained from the participants.

## Supporting information


Data S1:

Figure S1:

Figure S2:

## Data Availability

The original contributions presented in the study are included in the article, further inquiries can be directed to the corresponding author.
