# Supplementary material for: Gender‐specific outcomes of low‐dose computed tomography screening for lung cancer detection: A retrospective study in Chinese never‐smoker population
Source: Cancer Med. 2024 Sep 29;13(18):e70184. doi: 10.1002/cam4.70184 (PMC11439423; doi:10.1002/cam4.70184)
Supplement: Supplementary file 1 — Data S1: [file CAM4-13-e70184-s003.docx]

**Supplementary Meterial**

Below is a representative case involving a 62-year-old male with a BMI of 30.81 and no smoking history. He underwent initial lung LDCT screening on September 10, 2017, revealing a solid nodule in the right lower lobe and a mixed ground-glass opacity (mGGO) in the left upper lobe (Supplementary figure 1A). Subsequent video-assisted thoracoscopic surgery for radical resection of the right lower lobe lung cancer was performed on September 22, 2017. Pathological examination confirmed invasive adenocarcinoma with an EGFR mutation and lymph node reactive hyperplasia. The tumor size was 3.0*3.0*2.3 cm with vascular invasion and nerve involvement. Post-surgery genetic testing revealed a positive 19-DEL mutation. The postoperative diagnosis was stage IB invasive adenocarcinoma of the right lower lobe (pT2aN0M0, EGFR+). Adjuvant chemotherapy comprised pemetrexed (1000 mg d1) and cisplatin (75 mg d1-2) for four cycles.

Regular CT surveillance was conducted every three months post-surgery, transitioning to semi-annual scans, with no significant changes observed in the mGGO in the left upper lobe (Supplementary figure 1B). On May 20, 2020, he underwent video-assisted thoracoscopic surgery for radical resection of left upper lobe lung cancer, revealing invasive adenocarcinoma with predominant wall attachment and lymph node reactive hyperplasia. The nodule size was 1.0*0.7*0.4 cm. The postoperative diagnosis was stage IA invasive adenocarcinoma of the left upper lobe (pT1aN0M0), with no adjuvant therapy administered thereafter.


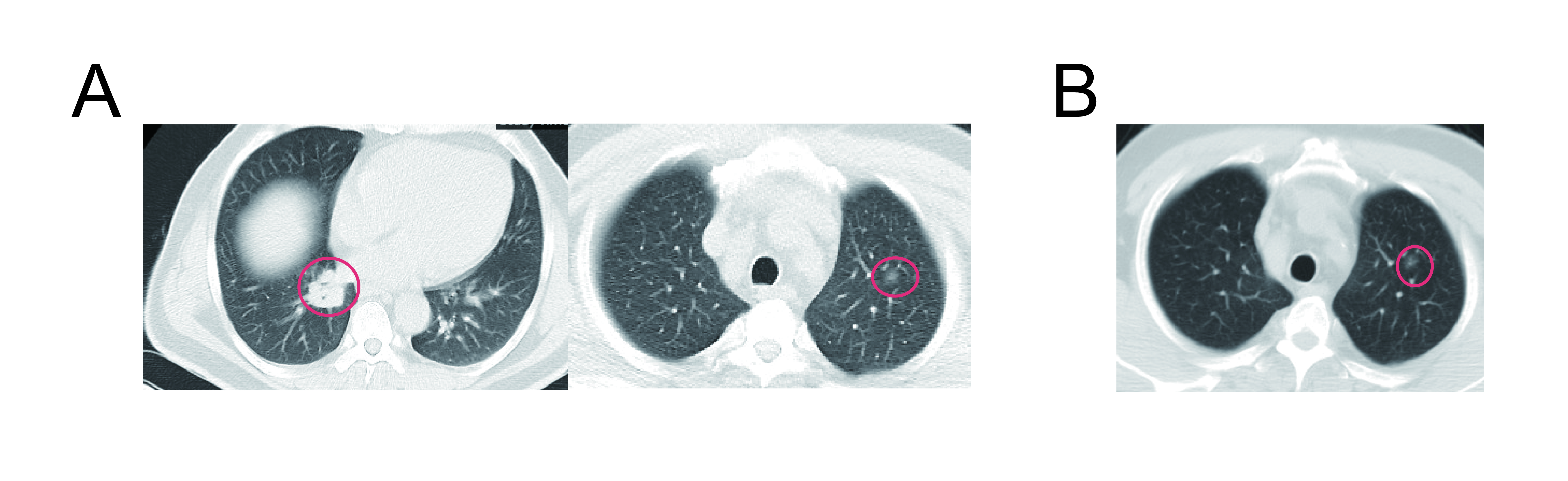


**Supplementary figure 1. Low-Dose Computed Tomography (LDCT) images depicting pulmonary nodules in a representative case with multiple nodules. (A)**. Images were obtained from the initial lung computed tomography (LDCT) scan conducted on September 10, 2017. Left panel: A solid nodule was detected in the lower lobe of the right lung, measuring 2.71 cm in maximum diameter, categorized as Lung-RADS 4B. Right panel: A mixed ground-glass opacity (mGGO) was observed in the upper lobe of the left lung, with a maximum diameter of 0.71 cm, classified as Lung-RADS 4A. **(B)**. Follow-up LDCT images were obtained on May 16, 2020, indicating no significant changes in the mGGO observed in the upper lobe of the left lung. Pulmonary nodules are highlighted in red circles.

**
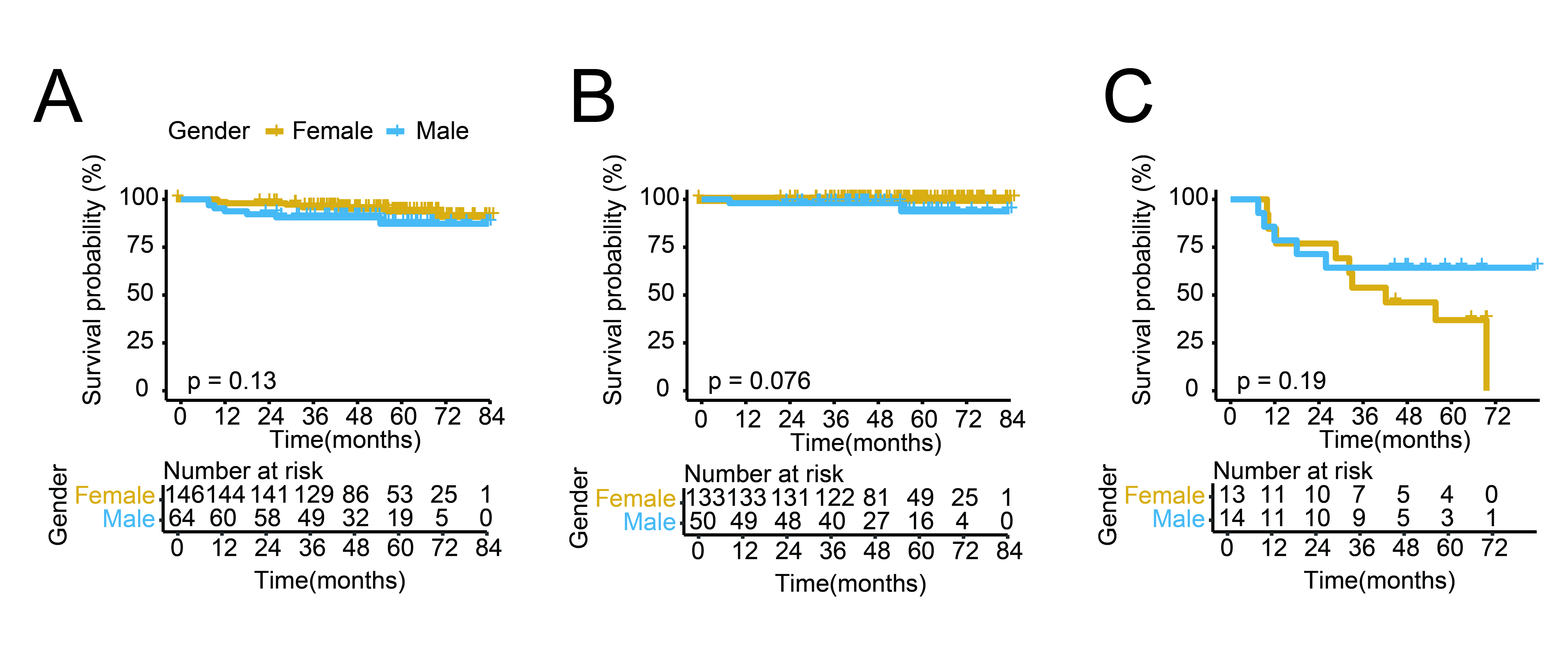
**

**Supplementary figure 2. Survival analysis of A) lung cancer, B) early-stage lung cancer, and C) advanced lung cancer in never-smoking females and males.**

**Supplementary Table 1. Characteristics of participants categorized with Lung-RADS 3 or 4 at initial LDCT screening.**

|  | **Total (%)** | **Male (%)** | **Female (%)** | | **P-value** | |
| --- | --- | --- | --- | --- | --- | --- |
| **Subjects** | 1119 | 597 | 522 | |  | |
| **Smoking status** |  |  |  |  | |  |
| Never-smokers | 880(78.64) | 363(60.80) | 517(99.04) | <0.001 | |  |
| Ever-smoker | 239(21.36) | 234(39.20) | 5(0.96) | <0.001 | |  |
| **Age range** |  |  |  | | 0.121 | |
| 20-29 y | 45(4.02) | 28(4.69) | 17(3.26) | |  | |
| 30-39 y | 112(10.01) | 59(9.88) | 53(10.15) | |  | |
| 40-49 y | 257(22.97) | 139(23.28) | 118(22.61) | |  | |
| 50-59 y | 348(31.10) | 200(33.50) | 148(28.35) | |  | |
| 60-69 y | 247(22.07) | 115(19.26) | 132(25.29) | |  | |
| 70-80 y | 110(9.83) | 56(9.38) | 54(10.34) | |  | |
| **BMI kg/m^2^** | 24.20±3.04 | 24.74±2.98 | 23.57±2.98 | | <0.001 | |
| **Lung-RADS category (at baseline LDCT)** |  |  |  | | 0.001 | |
| 3 | 674(60.23) | 371(62.14) | 303(58.05) | | 0.18 | |
| 4A | 156(13.94) | 83(13.90) | 73(13.98) | | 1 | |
| 4B | 55(4.92) | 33(5.53) | 22(4.21) | | 0.382 | |
| 4X | 234(20.91) | 110(18.43) | 124(23.75) | | 0.035 | |
| **Location of dominant nodule** |  |  |  | | 0.387 | |
| Right upper lobe | 268(23.95) | 137(22.95) | 131(25.10) | |  | |
| Right middle lobe | 118(10.55) | 56(9.38) | 62(11.88) | |  | |
| Right lower lobe | 269(24.04) | 140(23.45) | 129(24.71) | |  | |
| Left upper lobe | 207(18.50) | 121(20.27) | 86(16.48) | |  | |
| Left lower lobe | 249(22.25) | 138(23.12) | 111(21.26) | |  | |
| Trachea or main bronchus | 8(0.71) | 5(0.84) | 3(0.57) | |  | |
| **Nodule type** |  |  |  | | <0.001 | |
| Solid | 722(64.52) | 422(70.69) | 300(57.47) | | <0.001 | |
| Part-solid | 393(35.12) | 173(28.98) | 220(42.15) | | <0.001 | |
| Pure GGN | 0 | 0 | 0 | |  | |
| Cavitary | 4(0.36) | 2(0.34) | 2(0.38) | | 1 | |
| **Size of nodule at first detection (mm)** | 0.97±0.68 | 0.99±0.77 | 0.95±0.55 | | 0.237 | |
| **Follow-up** | 1006(89.90) | 544(91.12) | 462(88.51) | | 0.147 | |
| **Follow-up results** |  |  |  | | 0.006 | |
| Smaller or disappear | 167(16.60) | 98(18.01) | 69(14.94) | | 0.158 | |
| Unchanged | 497(49.40) | 287(52.76) | 210(45.45) | | 0.010 | |
| Growth | 8(0.80) | 3(0.55) | 5(1.08) | | 0.585 | |
| Received invasive biopsy | 334(33.20) | 156(28.68) | 178(38.53) | | 0.005 | |

**Supplementary table 2. Comparison of detection rates in ever-smokers versus non-smokers**

|  | **Total (%)** | **Ever-smoker (%)** | **Non-smoker (%)** | **P-value** |
| --- | --- | --- | --- | --- |
| **Subjects** | 42018 | 9423 | 32595 |  |
| **Nodules of Lung-RADS Score 3 and 4** | 1119(2.66) | 239(2.54) | 880(2.70) | 0.404 |
| **Lung cancer** | 258(0.61) | 46(0.49) | 212(0.65) | 0.089 |

**Supplementary table 3. Comparison of detection rates in male ever-smokers versus non-smokers**

|  | **Total (%)** | **Ever-smoker (%)** | **Non-smoker (%)** | **P-value** |
| --- | --- | --- | --- | --- |
| **Subjects** | 24581 | 9306 | 15275 |  |
| **Nodules of Lung-RADS Score 3 and 4** | 597(2.43) | 234(2.51) | 363(2.38) | 0.523 |
| **Lung cancer** | 109(0.44) | 65(0.70) | 44(0.29) | <0.001 |
| **Early-stage lung cancer** | 78(0.32) | 25(0.27) | 53(0.35) | 0.346 |

**Supplementary table 4. Comparison of detection rates in female ever-smokers versus non-smokers**

|  | **Total (%)** | **Ever-smoker (%)** | **Non-smoker (%)** | **P-value** |
| --- | --- | --- | --- | --- |
| **Subjects** | 17437 | 117 | 17320 |  |
| **Nodules of Lung-RADS Score 3 and 4** | 522(2.99) | 5(4.27) | 517(2.98) | 0.587 |
| **Lung cancer** | 149(0.85) | 2(1.71) | 147(0.85) | 0.614 |
| **Early-stage lung cancer** | 139(0.80) | 2(1.71) | 137(0.79) | 0.350 |

**Supplementary table 5. Characteristics of participants subjected to invasive diagnostic procedures.**

|  | | **Total (%)** | **Male (%)** | **Female (%)** | **P-value** |
| --- | --- | --- | --- | --- | --- |
| **Subjects** | | 334 | 156 | 178 |  |
| **Smoking status** |  |  |  |  | |
| Never-smokers | 263(78.74) | 88(56.41) | 175(98.31) | <0.001 | |
| Ever-smoker | 71(21.26) | 68(43.95) | 3(1.69) | <0.001 | |
| **Age range** | |  |  |  | 0.605 |
| 20-29 y | | 11(3.29) | 3(1.92) | 8(4.49) |  |
| 30-39 y | | 26(7.78) | 12(7.69) | 14(7.87) |  |
| 40-49 y | | 73(21.86) | 32(20.51) | 41(23.03) |  |
| 50-59 y | | 100(29.94) | 46(29.49) | 54(30.34) |  |
| 60-69 y | | 82(24.55) | 43(27.56) | 39(21.91) |  |
| 70-80 y | | 42(12.57) | 20(12.82) | 22(12.36) |  |
| **Nodule type** | |  |  |  | 0.007 |
| Solid | | 135(40.42) | 77(49.36) | 58(32.58) | 0.003 |
| Part-solid | | 195(58.38) | 77(49.36) | 118(66.29) | 0.003 |
| Cavitary | | 4(1.20) | 2(1.28) | 2(1.12) | 1 |
| **Number of LDCT screenings before biopsy** | | 2.19±1.18 | 2.22±1.30 | 2.17±1.06 | 0.767 |
| **Changes in nodules at baseline before biopsy** | |  |  |  | 0.025 |
| Direct surgery after baseline screening | | 89(26.65) | 46(29.49) | 43(24.16) | 0.330 |
| Unchanged | | 193(57.78) | 78(50.00) | 115(64.61) | 0.010 |
| Growth | | 47(14.07) | 28(17.95) | 19(10.67) | 0.080 |
| Nodule detected during follow-up | | 5(1.50) | 4(2.56) | 1(0.56) | 0.293 |
| **Diagnose** | |  |  |  | 0.001 |
| lung cancer | | 258(77.25) | 109(69.87) | 149(83.71) | 0.004 |
| metastatic carcinoma or lymphoma | | 4(1.20) | 0 | 4(2.25) |  |
| benign | | 67(20.06) | 43(27.56) | 24(13.48) | 0.002 |
| Unknown | | 5(1.50) | 4(2.56) | 1(0.56) |  |

**Supplementary table 6. Final diagnosis of benign nodules.**

|  | **Total (%)** | **Male (%)** | **Female (%)** |
| --- | --- | --- | --- |
| **Subjects** | 67 | 43 | 24 |
| Noninfectious inflammatory nodule | 19(28.36) | 11(25.58) | 8(33.33) |
| Nodules deposited by carbon | 12(17.91) | 8(18.60) | 4(16.67) |
| Hamartoma | 6(8.96) | 4(9.30) | 2(8.33) |
| Tuberculosis | 5(7.46) | 5(11.63) | 0 |
| Pulmonary cryptococcosis | 4(5.97) | 3(6.98) | 1(4.17) |
| AAH | 3(4.48) | 2(4.65) | 1(4.17) |
| Bronchial dysplasia | 3(4.48) | 2(4.65) | 1(4.17) |
| Pulmonary sclerosing pneumocytom | 2(2.99) | 1(2.33) | 1(4.17) |
| Glandular papilloma | 1(1.49) | 0 | 1(4.17) |
| Pulmonary isolation | 1(1.49) | 1(2.33) | 0 |
| Sarcoidosis | 1(1.50) | 0 | 1(4.17) |
| Unknown | 10(14.93) | 6(13.95) | 4(16.67) |

Noninfectious inflammatory nodule, comprising granulomatous inflammations (conditions confirmed to be non-tuberculous and free from fungal infections like Cryptococcus following staining and TB DNA tests) and chronic inflammatory nodules. AAH, atypical adenomatous hyperplasia. Unknown, surgeries performed at external hospitals, patients can only provide information that the nodule was benign, without detailed pathological identification.
