# Supplementary figures and images for: Gender‐specific outcomes of low‐dose computed tomography screening for lung cancer detection: A retrospective study in Chinese never‐smoker population
Source: Cancer Med. 2024 Sep 29;13(18):e70184. doi: 10.1002/cam4.70184 (PMC11439423; doi:10.1002/cam4.70184)

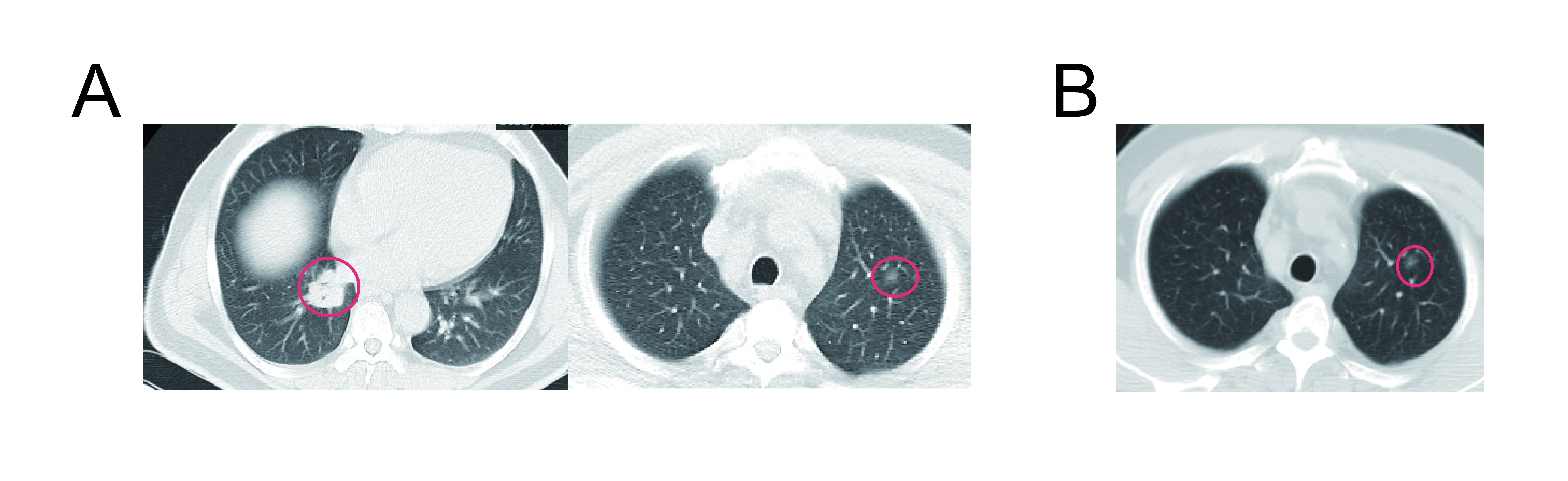

Supplement: Supplementary file 2 — Figure S1: [file CAM4-13-e70184-s001.jpg]

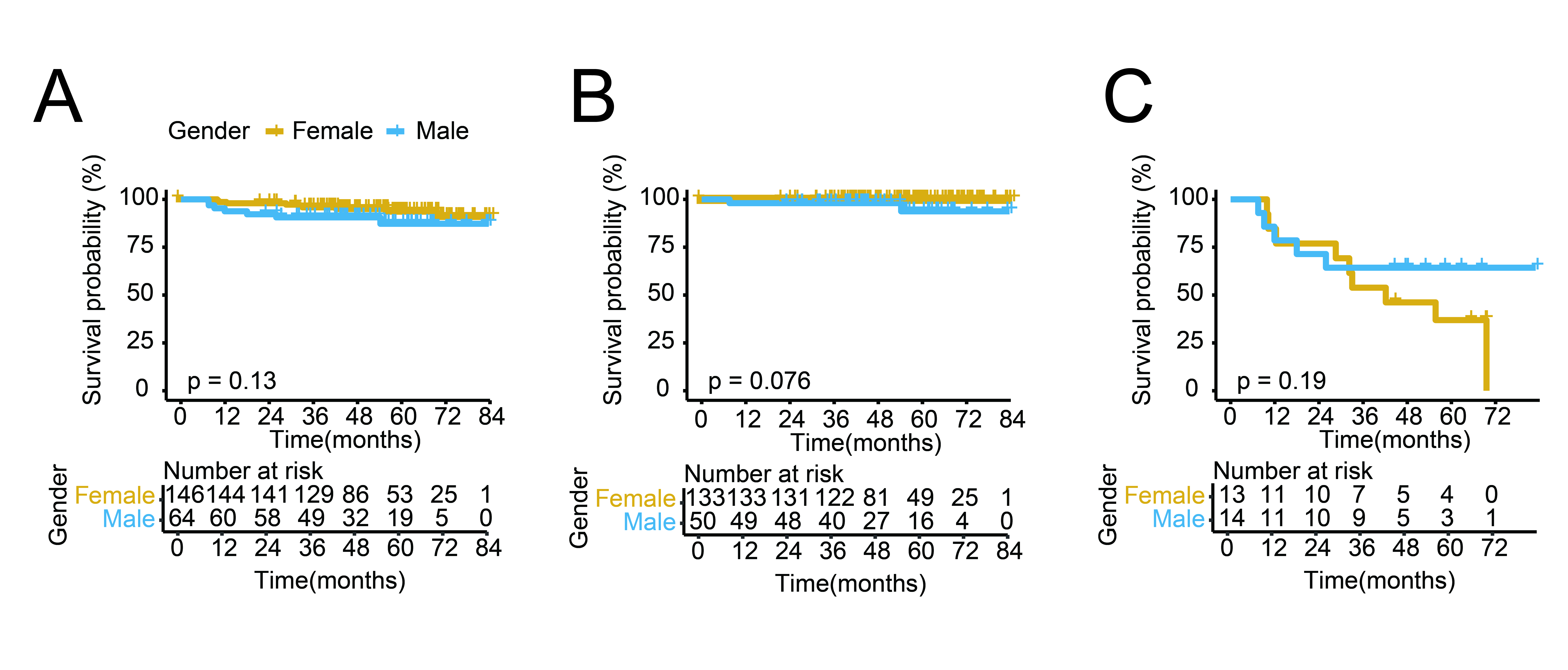

Supplement: Supplementary file 3 — Figure S2: [file CAM4-13-e70184-s002.jpg]
